# Characterization of microRNA and mRNA expression profiles in skin tissue between early-feathering and late-feathering chickens

**DOI:** 10.1186/s12864-018-4773-z

**Published:** 2018-05-25

**Authors:** Guijun Fang, Xinzheng Jia, Hua Li, Shuwen Tan, Qinghua Nie, Hui Yu, Ying Yang

**Affiliations:** 1 School of Life Science and Engineering, Foshan University, Foshan, 528231 Guangdong China; 2 College of Animal Science, South China Agricultural University, Guangzhou, 510642 Guangdong China; 3 Guangdong Tinoo’s Foods Limited Company, Qingyuan, 511827 Guangdong China

**Keywords:** microRNAs, mRNAs, Skin tissues, Late-feathering, Chicken

## Abstract

**Background:**

Early feathering and late feathering in chickens are sex-linked phenotypes, which have commercial application in the poultry industry for sexing chicks at hatch and have important impacts on performance traits. However, the genetic mechanism controlling feather development and feathering patterns is unclear. Here, miRNA and mRNA expression profiles in chicken wing skin tissues were analysed through high-throughput transcriptomic sequencing, aiming to understand the biological process of follicle development and the formation of different feathering phenotypes.

**Results:**

Compared to the N1 group with no primary feathers extending out, 2893 genes and 31 miRNAs displayed significantly different expression in the F1 group with primary feathers longer than primary-covert feathers, and 1802 genes and 11 miRNAs in the L2 group displayed primary feathers shorter than primary-covert feathers. Only 201 altered genes and 3 altered miRNAs were identified between the N1 and L2 groups (fold change > 2, q value < 0.01). Both sequencing and qPCR tests revealed that PRLR was significantly decreased in the F1 and L2 groups compared to the N1 group, whereas SPEF2 was significantly decreased in the F1 group compared to the N1 or L2 group. Functional analysis revealed that the altered genes or targets of altered miRNAs were involved in multiple biological processes and pathways related to feather growth and development, such as the Wnt signalling pathway, the TGF-beta signalling pathway, the MAPK signalling pathway, epithelial cell differentiation, and limb development. Integrated analysis of miRNA and mRNA showed that 14 pairs of miRNA-mRNA negatively interacted in the process of feather formation.

**Conclusions:**

Transcriptomic sequencing of wing skin tissues revealed large changes in F1 vs. N1 and L2 vs. N1, but few changes in F1 vs. L2 for both miRNA and mRNA expression. PRLR might only contribute to follicle development, while SPEF2 was highly related to the growth rate of primary feathers or primary-covert feathers and could be responsible for early and late feather formation. Interactions between miR-1574-5p/NR2F, miR-365-5p/JAK3 and miR-365-5p/CDK6 played important roles in hair or feather formation. In all, our results provide novel evidence to understand the molecular regulation of follicle development and feathering phenotype.

**Electronic supplementary material:**

The online version of this article (10.1186/s12864-018-4773-z) contains supplementary material, which is available to authorized users.

## Background

Early feathering (EF) and late feathering (LF) are sex-linked phenotypes that are usually determined in chickens at one day of age [1]. Based on the length of primary feathers and primary-convert feathers at hatching, the birds with the early-feathering phenotype are classified into two subtypes: the length of the primary feathers is more than 4.5 mm longer than that of the primary-convert feathers, or the length difference is in the range of 2 mm to 4.5 mm. The birds with the late-feathering phenotype are classified into four subtypes: the length of the primary feathers is longer than primary-coverts within 2 mm, the length of the primary feathers is shorter than that of the primary-covert feathers, the length of the primary feather and primary-covert feathers are the same, or the feathers are missing. In poultry production, the feather phenotype is utilized for sexing at one-day old.

However, the genetic mechanism of late-feathering is unclear. Previous studies have found that the K locus located on the Z chromosome was responsible for the feather phenotype, which was closely linked to the integration of endogenous retrovirus 21 (ev21) [2–5]. The latest investigations revealed that a tandem duplication of 176,324 bp linked to the K locus results in a partial duplication of Prolactin Receptor (PRLR) and Sperm Flagellar Protein 2 (SPEF2) [6]. Therefore, the function of PRLR and SPEF2 genes was destroyed in late-feathering birds, both of which were regarded as the major candidates impacting feather performance. PRLR is a ligand of PRL, which was involved in more than 300 separate biological activities, including reproduction, metabolism, water and electrolyte balance, growth and development, neurotransmission and behaviour, and immunoregulation [7]. PRLR was also reported to impact hair replacement in mice [8]. SPEF2, an unknown gene, plays a critical role in spermatogenesis and ciliary dyskinesia [9]. Subsequently, researchers focused on examining PRLR and SPEF2 gene expression between early and late feathering birds, and the results revealed that there were significantly different expression profiles in the skin tissue in some breeds [10, 11]. At the same time, many studies began to analyse the association between feather performance and economic production. The results showed that the existence of *ev*21 caused a reduction in egg production, an increase in infection by lymphoid leucosis virus and an increase in the mortality rate [12]. These negative effects indicated that it was necessary to exploit the genetic mechanism of various feather phenotypes and to clarify how the late feathers are formatted.

MicroRNA (miRNA) is a class of non-coding small RNAs with a length of approximately 22 nt, which plays its post-transcription regulation roles mainly by degrading mRNA or inhibiting the translation process [13]. MiRNA is involved in almost all biological processes [14, 15]. Recent research has revealed that miRNA also participated in follicle development and feather formation [16, 17]. These results indicated that it would be helpful to perform genome-wide transcriptome analysis on miRNA and mRNA in chicken skin tissues, aiming to identify the major genes controlling feather formation and feather phenotypes. Our results would highlight novel genes or pathways to better understand the molecular mechanism of early and late feathering in birds.

## Results

### Overview of the sequencing data

**Fig. 1:**
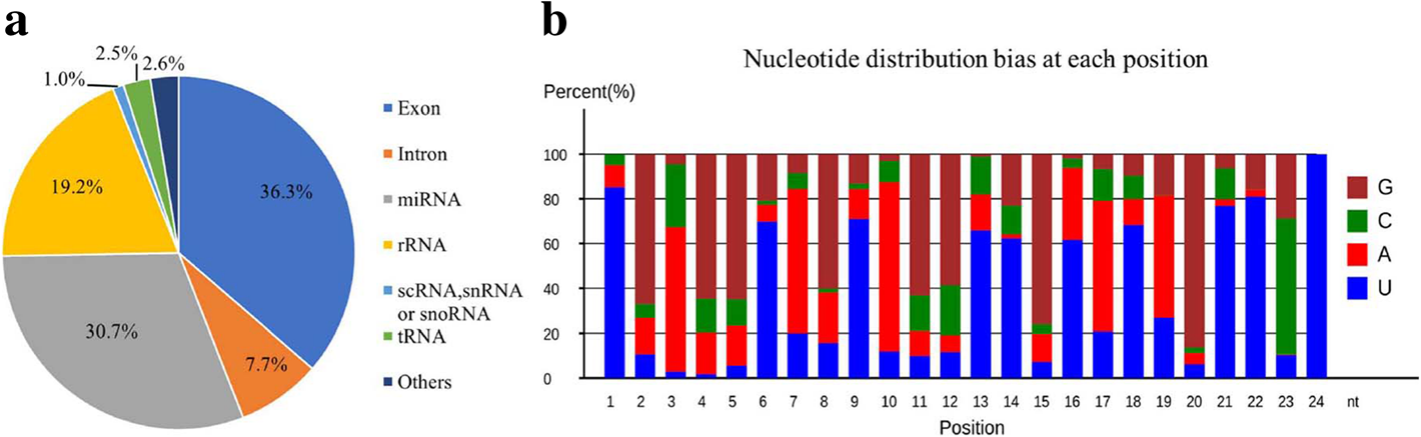
Characterization of small RNA sequencing data. **a** The distribution of raw reads mapped to the chicken genome. **b** The nucleotide bias of small RNA reads

The small and long RNA sequencing provided by Illumina technology generated an average of 16–19 million single-end high-quality reads and 65–69 million paired-end high-quality reads from each sample. For small RNA sequencing data, 21–24 nt length reads were the most abundant reads, and genome mapping results showed that approximately 44.0% high-quality reads were mapped to exon and intron regions, approximately 30.7% high-quality reads were mapped to annotated miRNAs, and the remaining 25.3% were mapped to other small RNAs (Fig. 1a). Characterization of these 21–24 nt long small RNAs reveals an obvious bias for uracil (U) at their 5′ ends and 3′ ends (Fig. 1b). For long RNA sequencing data, more than 81.9% reads were mapped to the chicken genome in the paired-end model, yielding approximately 56 million reads for gene expression analysis.

### Differentially expressed miRNAs in skin tissue from early-feathering and late-feathering birds

Four hundred and fifty-eight miRNAs were detected in one-day-old chicken wing skin tissues, which accounted for approximately half of the miRNAs reported from chickens. Thirty-three miRNAs were significantly differently expressed between each group (Table 1). Compared to late-feathering birds with no primary feathers extending out (N1 group), the expression of 31 miRNAs was significantly altered in the early-feathering birds, including 11 upregulated miRNAs and 20 downregulated ones. Compared to another type of late feathering birds with primary feathers shorter than primary-covert feathers (L2 group), only 3 miRNAs were different in the early-feathering birds (F1 group). However, there were still 11 differentially expressed miRNAs between the two subtypes of late-feathering birds. Interestingly, all 11 altered miRNAs overlapped between F1 vs. N1 and L2 vs. N1. Gga-miR-31-5p was the only miRNA that overlapped among all the possible comparisons, which was downregulated in F1 vs. N1 and F1 vs. L2, while upregulated in N1 vs. L2.

**Table 1:** Differentially expressed miRNAs among early and late feathering groups

	miRNA	Log_2_FC	q value	Changes
F1 vs. N1	gga-miR-217-5p	2.76	7.62E-07	Up
	gga-miR-6651-5p	2.61	2.59E-03	Up
	gga-miR-216a	2.47	2.40E-03	Up
	gga-miR-3538	1.88	3.58E-03	Up
	gga-miR-1729-5p	1.87	5.62E-03	Up
	gga-miR-216b	1.86	5.81E-03	Up
	gga-miR-383-5p	1.66	7.93E-03	Up
	gga-miR-199b	1.60	2.59E-03	Up
	gga-miR-183	1.50	1.02E-03	Up
	gga-miR-146b-5p	1.28	4.19E-08	Up
	gga-miR-181a-5p	1.03	1.35E-04	Up
	gga-miR-1329-5p	−1.30	8.72E-03	Down
	gga-miR-193a-5p	−1.35	1.03E-04	Down
	gga-miR-1552-5p	−1.37	3.97E-04	Down
	gga-miR-130a-5p	−1.44	8.86E-03	Down
	gga-miR-200a-5p	−1.55	6.21E-06	Down
	gga-miR-146a-5p	−1.79	8.97E-11	Down
	gga-miR-449a	−2.03	5.94E-03	Down
	gga-miR-200b-5p	−2.16	2.59E-08	Down
	gga-miR-130b-5p	−2.30	2.09E-06	Down
	gga-miR-365-2-5p	−2.32	4.82E-07	Down
	gga-miR-34a-5p	−2.36	2.04E-03	Down
	gga-miR-6566-5p	−2.90	1.26E-03	Down
	gga-miR-1663-5p	−2.90	1.35E-04	Down
	gga-miR-204	−3.03	4.17E-10	Down
	gga-miR-211	−3.03	4.17E-10	Down
	gga-miR-1674	−3.22	2.65E-04	Down
	gga-miR-2954	−3.36	1.73E-04	Down
	gga-miR-1759-5p	−3.44	1.73E-04	Down
	gga-miR-31-5p	−3.52	2.12E-32	Down
	gga-miR-1574-5p	−4.18	1.35E-04	Down
F1 vs. L2	gga-miR-1c	2.69	8.53E-04	Up
	gga-miR-499-5p	2.64	1.73E-03	Up
	gga-miR-31-5p	−1.14	1.95E-04	Down
L2 vs. N1	gga-miR-199b	1.92	2.15E-04	Up
	gga-miR-6651-5p	1.95	9.19E-03	Up
	gga-miR-31-5p	−2.28	3.36E-16	Down
	gga-miR-200b-5p	−1.78	1.31E-08	Down
	gga-miR-365-2-5p	−1.99	4.74E-06	Down
	gga-miR-211	−2.21	9.36E-06	Down
	gga-miR-204	−2.21	9.36E-06	Down
	gga-miR-200a-5p	−1.21	1.40E-05	Down
	gga-miR-146a-5p	−1.07	7.10E-04	Down
	gga-miR-1674	−2.79	1.20E-03	Down
	gga-miR-1552-5p	−1.02	4.91E-03	Down

Differentially expressed miRNA of each comparison were selected with a setting of q value < 0.01 and fold change > 2

### Differentially expressed mRNAs in skin tissue between early-feathering and late-feathering birds

**Fig. 2:**
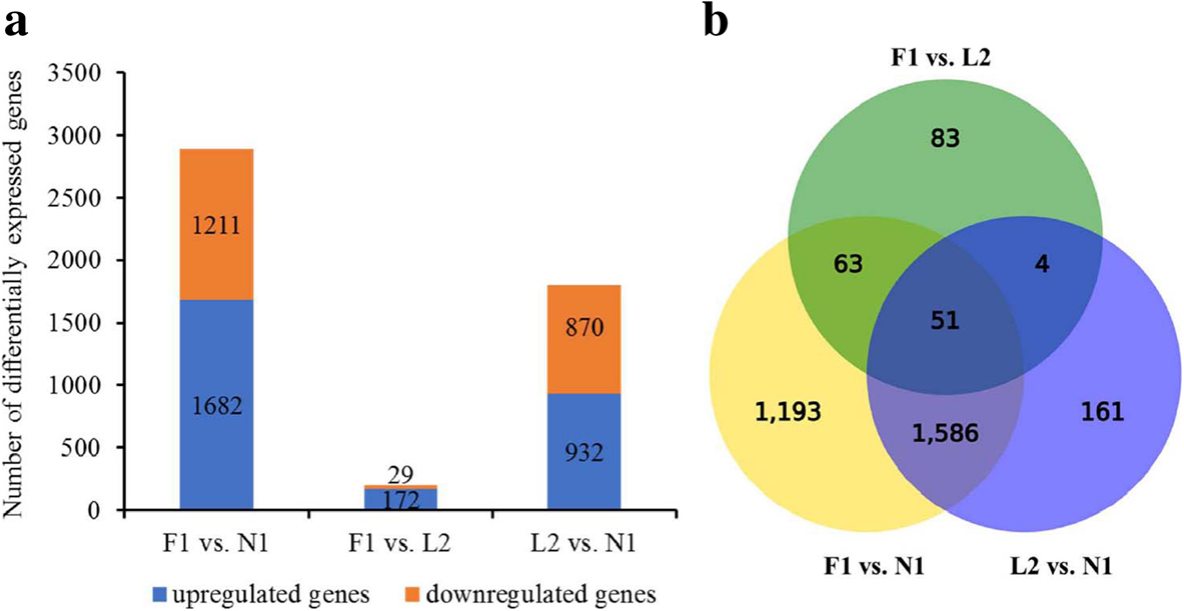
The differentially expressed genes in each group. **a** The numbers of upregulated and downregulated genes in three pairwise comparisons. **b** The numbers of differentially expressed genes overlapped in three pairwise comparisons. DEGs of each comparison were selected with a setting of q value < 0.01 and fold change > 2

The analysis of differentially expressed genes revealed a significant difference in skin tissues between early-feathering and late-feathering birds. There were 1802 significantly differently expressed mRNAs between the late-feathering L2 group and the N1 group, including 932 upregulated and 870 downregulated genes. In addition, 2893 differently expressed mRNAs were identified between the late-feathering N1 group and the early-feathering F1 group, including 1682 upregulated and 1211 downregulated genes in the F1 group. However, there were only 201 differentially expressed mRNAs between the early-feathering F1 group and the late-feathering L2 group, including 172 upregulated and 29 downregulated genes (Fig. 2a). Among these differentially expressed genes, 114 genes overlapped between the early-feathering F1 group and the late-feathering N1 or L2 group. However, 1637 genes overlapped between F1 vs. N1 and L2 vs. N1, and 55 genes overlapped between F1 vs. L2 and L2 vs. N1. In all, there were 51 genes overlapping among each group (Fig. 2b).

Based on functional annotation, the expression patterns of most feather-follicle-development related genes, except EGF and SPEF2, were similar in the F1 group with the primary feathers longer than primary-covert feathers and the L2 group with primary feathers shorter than primary-covert feathers, compared to the N1 group with no feathers grown out. The results indicated that these genes might generally contribute to feather growth and development, without special roles in primary or primary-covert feathers. Interestingly, PRLR and SPEF2 were reported to be candidate genes related to the late-feathering phenotype [6]. In this study, the expression level of PRLR was significantly lower in the F1 group and the L2 group than in the N1 group, but there was no significant change between the F1 and L2 groups (Table 2). However, SPEF2 had a significantly lower expression level in the F1 group compared to the N1 or L2 group, suggesting that the expression pattern of SPEF2 was strongly associated with the early- and late-feathering phenotypes. In addition, EGF exhibited a completely similar expression pattern to SPEF2, which indicated that EGF might be a novel candidate gene associated with early- and late-feathering phenotypes.

**Table 2:** The expression profiles of genes related to feather follicle development

F1 vs. N1			L2 vs. N1			F1 vs. L2		
Gene	Log_2_FC	q value	Gene	Log_2_FC	q value	Gene	Log_2_FC	q value
SHH	2.8	1.3E-04	SHH	2.5	2.8E-04	SHH	0.2	7.3E-01
CRABP1	2.4	2.9E-21	CRABP1	1.5	5.5E-04	CRABP1	0.8	6.9E-02
BMP7	1.7	1.5E-23	BMP7	1.6	3.0E-22	BMP7	0.1	5.5E-01
BMP2	1.6	2.8E-12	BMP2	1.6	6.6E-13	BMP2	0.1	9.0E-01
WNT3A	1.2	1.6E-04	WNT3A	0.8	1.3E-02	WNT3A	0.3	3.8E-01
PRLR	−1.1	9.1E-07	PRLR	−0.7	1.5E-06	PRLR	−0.4	3.1E-01
FGF2	−1.4	3.3E-09	FGF2	−1.1	3.6E-05	FGF2	−0.3	4.9E-01
PDGFA	−1.9	2.4E-23	PDGFA	−1.1	5.2E-08	PDGFA	−0.7	1.6E-02
FGFR	−1.6	3.0E-18	FGFR	−1.3	1.3E-16	FGFR	−0.3	4.2E-01
EGF	−2.3	2.7E-04	EGF	−0.1	9.0E-01	EGF	−2.1	1.0E-07
SPEF2	−2.8	2.0E-12	SPEF2	−0.5	1.1E-01	SPEF2	−2.0	9.4E-07
TGFB1	−1.0	2.7E-08	TGFB1	− 1.0	3.6E-07	TGFB1	0.1	9.1E-01

### Validation of differentially expressed miRNAs and mRNAs

**Fig. 3:**
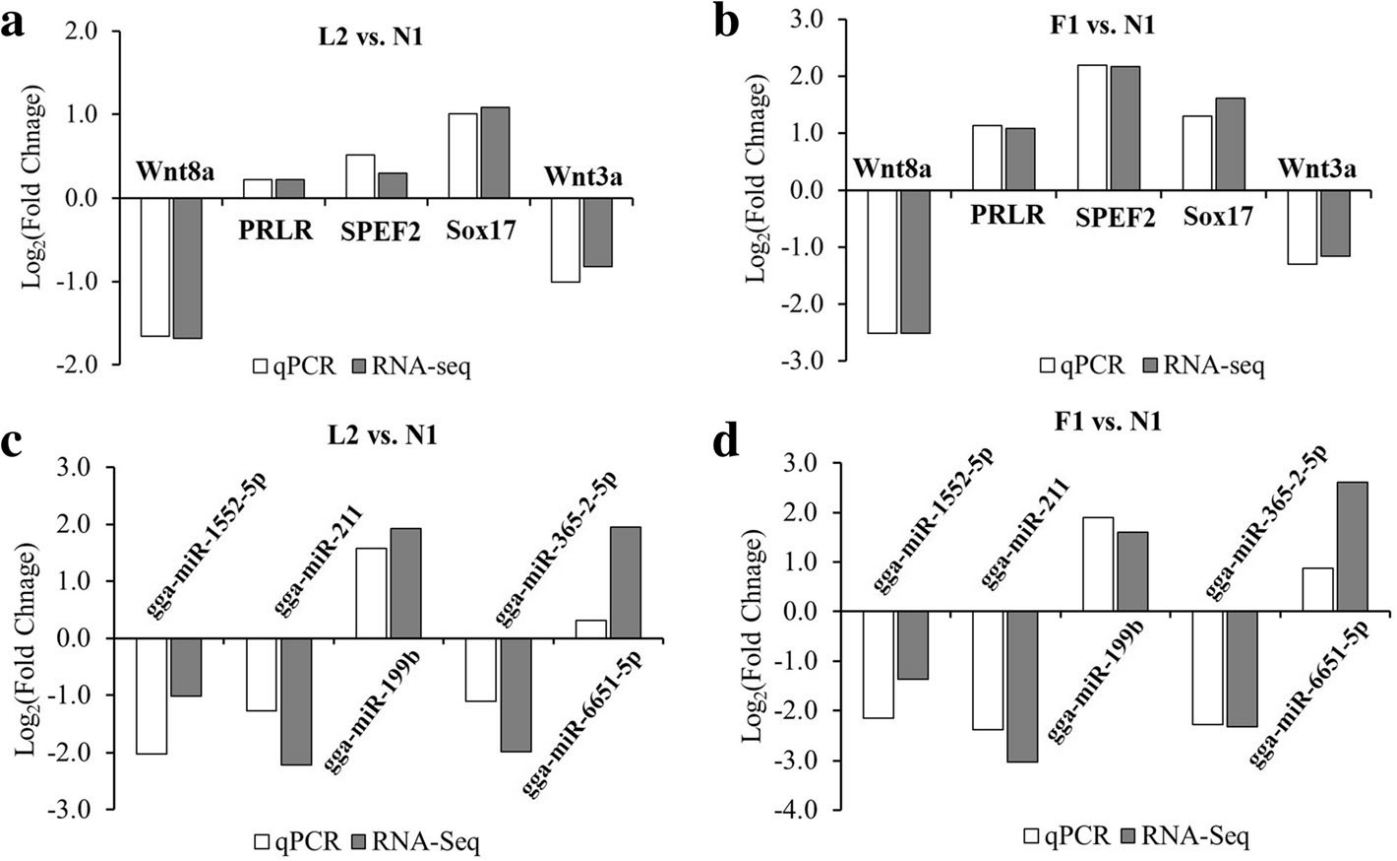
Validation of miRNA and mRNA expression profiles by qPCR. **a** The expression pattern of five genes were tested between L2 and N1 group. **b** The expression pattern of five genes were tested between F1 and N1 group. **c** The expression pattern of five miRNAs were tested between L2 and N1 group. **d** The expression pattern of five miRNAs were tested between F1 and N1 group. U6 and β-actin were used as reference genes for miRNA and mRNA testing, respectively. Fold change values of qPCR were calculated using the comparative 2^–∆∆CT^ (∆∆Ct = ∆Ct (target gene) – ∆Ct (reference gene)) from at least three independent experiments. Fold change values of sequencing data were calculated using the DESeq2 system

To validate the sequencing data, qPCR was employed to test the relative expression profiles of 5 differentially expressed miRNAs and mRNAs among each group in the same skin tissues. The results showed that the alteration of these miRNAs and mRNAs from the RNA-seq data were confirmed by qPCR, and their altered expression patterns among different groups were well-matched with the RNA-seq data, which guaranteed the accuracy of subsequent functional analysis (Fig. 3).

### Functional analysis of differentially expressed miRNAs and mRNAs

To analyse the molecular function of these differentially expressed miRNAs, both of RNA hybrid and miRanda systems were utilized to improve the prediction of miRNA targets, resulting in 2665 targets potentially regulated by total 33 miRNAs. Functional enrichment analysis revealed that 22 differentially expressed miRNAs mainly contributed to several key molecular processes, including cell differentiation (24 genes), cell fate commitment (9 genes), the Wnt signalling pathway (11 genes), epithelial cell differentiation (8 genes), peptidyl-tyrosine autophosphorylation (7 genes), limb development (7 genes), embryonic limb morphogenesis (6 genes) and negative regulation of osteoblast differentiation (6 genes), of which the latter 6 processes are highly related to skin and feather development (Table 3). In addition, gga-miR-34a-5p and gga-miR-365-2-5p nearly participated in nearly all these processes by targeting 12 and 11 genes, respectively. All these results suggested that several altered miRNAs played important roles in the control of follicle development.

**Table 3:** Functional enrichment of altered miRNAs

Term	Count	*p* value	miRNA
Cell differentiation	24	0.01	gga-miR-130b-5p, gga-miR-130a-5p, gga-miR-365-2-5p, gga-miR-193a-5p, gga-miR-449a, gga-miR-34a-5p, gga-miR-1674, gga-miR-31-5p, gga-miR-383-5p, gga-miR-1759-5p, gga-miR-2954
Cell fate commitment	9	0.01	gga-miR-2954, gga-miR-34a-5p, gga-miR-193a-5p gga-miR-1663-5p, gga-miR-211, gga-miR-204, gga-miR-449a, gga-miR-6566-5p
Wnt signalling pathway	11	0.05	gga-miR-2954, gga-miR-211, gga-miR-204, gga-miR-6651-5p, gga-miR-6566-5p, gga-miR-34a-5p, gga-miR-365-2-5p
Epithelial cell differentiation	8	0.05	gga-miR-6651-5p, gga-miR-216a, gga-miR-365-2-5p, gga-miR-34a-5p, gga-miR-130b-5p, gga-miR-1552-5p
Peptidyl-tyrosine autophosphorylation	7	0.07	gga-miR-365-2-5p, gga-miR-1674, gga-miR-6651-5p, gga-miR-34a-5p, gga-miR-449a
Limb development	7	0.07	gga-miR-34a-5p, gga-miR-1574-5p, gga-miR-2954, gga-miR-365-2-5p, gga-miR-193a-5p, gga-miR-211, gga-miR-204
Embryonic limb morphogenesis	6	0.08	gga-miR-34a-5p, gga-miR-193a-5p, gga-miR-204, gga-miR-211, gga-miR-1552-5p, gga-miR-200b-5p
Negative regulation of osteoblast differentiation	6	0.09	gga-miR-34a-5p, gga-miR-193a-5p, gga-miR-365-2-5p, gga-miR-1663-5p

The 2893 differentially expressed genes between the F1 and N1 groups were significantly enriched in 17 signalling pathways, including the TGF-beta signalling pathway (24 genes), Tyrosine tyrosine metabolism (11 genes), the MAPK signalling pathway (47 genes) and the Wnt signalling pathway (28 genes; Table 4). However, 1802 differentially expressed genes between the L2 and N1 groups were significantly enriched in 12 signalling pathways, which completely overlapped with the results of the comparison of F1 and the N1 group, except for the PPAR signalling pathway and nicotinate and nicotinamide metabolism (Table 5). Also, even in the same enriched pathway, there were obvious difference between the comparisons of F1 vs. N1 and L2 vs. N1. For example, there were 24 altered genes significantly enriched in TGF-beta signalling pathway from the comparison of F1 vs. N1, and whereas only 13 altered genes were enriched in the comparison of L2 vs. N1.

**Table 4:** Pathway enrichment analysis on differentially expressed genes between F1 and N1 group

KEGG pathway	Genes count	Percent	*p* value
Cell adhesion molecules (CAMs)	46	1.6	3.40E-10
Cytokine-cytokine receptor interaction	60	2.1	5.80E-10
Neuroactive ligand-receptor interaction	78	2.7	4.10E-08
Focal adhesion	53	1.8	2.50E-05
Calcium signalling pathway	47	1.6	5.50E-05
ECM-receptor interaction	26	0.9	1.40E-04
Melanogenesis	29	1	2.90E-04
TGF-beta signalling pathway	24	0.8	1.20E-03
Vascular smooth muscle contraction	29	1	2.60E-03
Adherens junction	20	0.7	1.10E-02
Tyrosine metabolism	11	0.4	2.20E-02
MAPK signalling pathway	47	1.6	2.50E-02
Gap junction	21	0.7	2.70E-02
Adrenergic signalling in cardiomyocytes	29	1	2.90E-02
Wnt signalling pathway	28	1	4.40E-02
Glycine, serine and threonine metabolism	11	0.4	4.90E-02

**Table 5:** Pathway enrichment analysis on differentially expressed genes between L2 and N1 group

KEGG pathway	Genes count	Percent	*p* value
Cell adhesion molecules (CAMs)	32	1.8	9.60E-09
Neuroactive ligand-receptor interaction	45	2.5	8.80E-05
Melanogenesis	20	1.1	5.20E-04
Cytokine-cytokine receptor interaction	30	1.7	6.80E-04
ECM-receptor interaction	15	0.8	7.60E-03
PPAR signalling pathway	12	0.7	2.10E-02
Tyrosine metabolism	8	0.4	2.30E-02
Vascular smooth muscle contraction	17	0.9	2.70E-02
Calcium signalling pathway	24	1.3	2.90E-02
Nicotinate and nicotinamide metabolism	7	0.4	3.70E-02
Focal adhesion	26	1.4	4.00E-02
TGF-beta signalling pathway	13	0.7	4.40E-02

### miRNA-mRNA interaction and signalling pathways related to feather development

MiRNAs have roles in biological regulation mainly through interactions between miRNA and its target mRNA, which commonly causes downregulated mRNA expression. Here, integrated analysis of both miRNA and mRNA expression profiles in the same skin tissues was performed to characterize miRNA-mRNA pairs with inverse expression levels and analyse their biological processes. For the comparison between the F1 and N1 groups, 14 pairs of miRNA-mRNA with negative regulation were identified (Table 6). Among these, gga-miR-216a, gga-miR-365-2-5p and gga-miR-130b-5p participate in epithelial cell differentiation through negatively regulating the expression of KRT6A, UPK1B and TCF21, respectively. Gga-miR-1574-5p and gga-miR-365-2-5p are involved in limb development by negatively regulating NR2F2 and PDLIM4. Gga-miR-193a-5p impacts embryonic limb morphogenesis by negatively regulating PRRX1. For the comparison between the L2 and N1 groups, only 5 pairs of miRNA-mRNA were identified, which mainly participate in cell differentiation, limb development and negative regulation of osteoblast differentiation (Table 7). In all, these miRNA-mRNA pairs cooperatively controlled multiple embryo development processes related to wing and follicle development.

**Table 6:** MiRNA-mRNA pairs with inverse expression relationship between the F1 and N1 groups

Gene	Log_2_FC	miRNA	Log_2_FC	Biological process
ERG	1.6	gga-miR-130b-5p	−2.3	Cell differentiation
TEC	1.2	gga-miR-1674	−3.2	
JAK3	1.3	gga-miR-365-2-5p	−2.3	
ERBB4	1.4	gga-miR-34a-5p	−2.4	Cell fate commitment
GAP43	1.7	gga-miR-449a	−2.0	
KRT6A	−1.8	gga-miR-216a	2.5	Epithelial cell differentiation
UPK1B	1.9	gga-miR-365-2-5p	−2.3	
TCF21	2.6	gga-miR-130b-5p	−2.3	
NR2F2	1.6	gga-miR-1574-5p	−4.2	Limb development
PDLIM4	1.6	gga-miR-365-2-5p	−2.3	
PRRX1	1.1	gga-miR-193a-5p	−1.4	Embryonic limb morphogenesis
CDK6	1.4	gga-miR-365-2-5p	−2.3	Negative regulation of osteoblast differentiation
LIMD1	1.4	gga-miR-365-2-5p	−2.3	
HDAC7	1.4	gga-miR-1663-5p	−2.9	
JAK3	1.3	gga-miR-365-2-5p	−2.3	Peptidyl-tyrosine autophosphorylation
TEC	1.2	gga-miR-1674	−3.2	
ERBB4	1.4	gga-miR-34a-5p	−2.4

**Table 7:** MiRNA-mRNA pairs with inverse expression relationship between the L2 and N1 groups

Gene	Log_2_FC	miRNA	Log_2_FC	Biological process
JAK3	1.34	gga-miR-365-2-5p	−1.99	Cell differentiation
PDLIM4	1.20	gga-miR-365-2-5p	−1.99	Limb development
LIMD1	1.14	gga-miR-365-2-5p	−1.99	Negative regulation of osteoblast differentiation
CDK6	1.12	gga-miR-365-2-5p	−1.99	
UPK1B	1.14	gga-miR-365-2-5p	−1.99	Epithelial cell differentiation

## Discussion

The feathering phenotype is a sex-linked trait and can be utilized to distinguish the sexes at hatching based on differences in the rate of feather growth between primary and primary-covert feathers. To understand the molecular mechanism controlling feather development, chicken wing skin tissues at hatching were used for transcriptome analysis from three different groups: primary feathers longer than primary-covert feathers, or no feathers, or primary feathers shorter than primary-covert feathers. In this study, compared to the N1 group, the F1 and L2 groups exhibited faster growth in feather development, and the main differences between F1 and L2 were the growth rate in primary and primary-covert feathers. In addition, the N1 and L2 groups exhibited the late-feathering phenotype, and the F1 group exhibited the early-feathering phenotype. Thus, the candidate genes or miRNAs related to feather development would be identified through comparing the expression profiles among the F1, N1 and L2 groups.

Transcriptome and expression profiling analysis revealed large changes in F1 vs. N1 (2893 DEGs) and L2 vs. N1 (1802 DEGs) but few changes in F1 vs. L2 (201 DEGs). Similar patterns were also observed for the miRNA expression profiles. Among these genes, 1637 genes overlapped between F1 vs. N1 and L2 vs. N1, indicating that these genes were mainly responsible for feather growth and development with no impact on feather types and growth rate. To analyse the difference between early and late feathering groups, 114 genes overlapped between F1 vs. N1 and F1 vs. L2, suggesting that these genes were mainly responsible for feather rate and feather phenotype. These results also revealed a larger number of genes controlling feather development from follicles but a smaller number of genes controlling feather growth rate and feather phenotype. This result is consistent with current genetic mapping of early and late feathering phenotypes. The late feathering phenotype was shown to be controlled by the K allele located on the Z chromosome [2]. Most studies observed that provirus ev21 was closely associated with the late feathering locus [3, 4]. Recent reports revealed a tandem duplication close to the K allele, resulting in the partial duplication of the PRLR and SPEF2 genes [6]. Subsequently, PRLR and SPEF2 were regarded as candidate genes impacting the early feathering and late feathering phenotypes [10, 11, 18]. PRLR is a member of the cytokine receptor family and is closely related to the growth hormone receptor [19]. PRLR knockout investigations in mice revealed a change in the timing of hair follicle cycling events, which indicated a high association between PRL signalling and follicle development [8]. In this study, we also focused on the expression profiles among the F1, L2 and N1 groups. The PRLR gene was 2.1-fold and 1.6-fold lower in the F1 group and the L2 group compared to the N1 group, but no significant change was seen between the F1 and L2 groups. This result suggested that PRLR suppression might contribute to follicle development but has no huge impacts on the growth rate of primary feathers or primary-covert feathers. Thus, the transcriptome data from skin tissues indicated that the feather phenotype was not determined by the PRLR gene. Previous studies showed that the expression level of PRLR in the wing skin of late feathering chickens was 1.7-fold higher than that in the early feathering type of Wenchang chickens at hatching, while a similar investigation in Suqin green-egg-shelled chickens showed no change between early and late feathering types [10, 11]. However, the expression profiles of different types of early feathering birds or late feathering birds were not compared, which should be undertaken in the future. Differently to PRLR, SPEF2 had 7.0-fold and 4.0-fold lower expression in the F1 group compared to the N1 and L2 groups, both of which were of late feathering birds, with no significant difference between each other. The same expression pattern was verified in other studies [10]. It is reasonable to predict that SPEF2 is strongly related to early and late feather formation.

The formation of feathers begins in the follicles. The feather filament grows out of the follicle via epithelial cell proliferation and differentiation. Complex feather development is controlled by multiple genes involved in gene regulation and signalling transition. Through comparing F1 or L2 to the N1 group, many differentially expressed genes and miRNAs were identified in our research that might contribute to the biological processes of feather development. Functional annotation revealed genes enriched in cytokine-cytokine receptor interaction and neuroactive ligand-receptor interaction, which is related to PRLR signalling. They were also enriched in growth-related pathways, including the Wnt signalling pathway, the TGF-beta signalling pathway and the MAPK signalling pathway. Additionally, functional analyses of targets of miRNA uncovered 22 differentially expressed miRNAs involved in epithelial cell differentiation and cell fate commitment, as well as embryonic limb development and morphogenesis, which have been interpreted in the linkage of limb development, skin cell differentiation, follicle development and feather formation [20].

MiRNAs, as post-transcriptional factors and key regulators of transcriptional gene networks, play an important role in hair cell generation [21, 22]. Several miRNAs, including the miR-183 family, miR-96, miR-15, miR-99, miR-100, miR-125, and miR-133, all might contribute to hair cell development and maintenance [23–26]. In domestic animals such as duck, goats and sheep, miRNAs play important roles in the development of follicles [27–29]. Here, miRNA-mRNA interaction pairs were analysed to identify the key factors impacting feather formation. Fourteen pairs between the F1 and N1 groups and 5 pairs between the L2 and N1 groups were negatively regulated in the gene regulatory network. We found that NR2F was inhibited by gga-miR-1574-5p in chicken skin tissue to impact limb development. NR2F was reported to be a key factor controlling hair cell reprogramming [30]. Another study found that miR-302 could increase the reprogramming efficiency of hair follicle cells via repression of NR2F2 [31]. In this study, miR-302 exhibited no significant change, indicating that different miRNA-mRNA regulatory interactions controlled feather formation. Gga-miR-365-2-5p was higher in the N1 group compared to the F1 and L2 groups, suggesting that it might inhibit feather growth and development by downregulating the expression of JAK3, PDLIM4, LIMD1, CDK6 and UPK1, which are involved in epithelial cell differentiation and limb development. Interestingly, JAK inhibition was shown to be essential to hair regrowth [32], and CDK6 plays important roles in hair cell differentiation through controlling the cell cycle [33, 34]. All these results revealed that miR-365-5p might have an important effect on feather or hair generation via JAK3 and CDK6 signals.

## Conclusions

Transcriptome sequencing of wing skin tissues uncovered different expression profiles among chickens with various feather phenotypes at hatching, resulting in large changes in both miRNA and mRNA expression in F1 vs. N1 and L2 vs. N1 but few changes in F1 vs. L2. PRLR might only contribute to follicle development, while SPEF2 was highly related to the growth rate of primary feathers or primary-covert feathers and could be responsible for early and late feather formation. miR-1574-5p/NR2F, miR-365-5p/JAK3 and miR-365-5p/CDK6 interactions played important roles in hair or feather formation. In conclusion, our results provide new insights to understand the molecular regulation of follicle development and the feathering phenotype.

## Methods

### Animals

The wing skin tissues were collected in RNAlater (Ambion, Thermo Fisher), solution from 18 Qingyuan partridge chickens at one-day old, provided by the Guangdong Tinoo’s Foods Co., Ltd., including three groups with 6 individuals in each: later-feathering L2 group (L2) with primary feathers shorter than primary-covert feathers, later-feathering N1 group (N1) with no primary feathers extending out, and early-feathering F1 group (F1) with primary feathers longer than primary-covert feathers more than 2 mm.

### RNA libraries preparation and sequencing

Nine small RNA libraries and 9 long RNA libraries were prepared with three replicates in each group. Briefly, total RNA was isolated from each skin tissue (approximately 80 mg) using TRIzol reagent (Invitrogen, USA) according to the manufacturer’s protocol. The RNA purity and quantity were tested using a NanoDrop 2000 spectrophotometer based on the OD230/260/280 value. Then, DNA treatment was performed using a DNA-free kit (Ambio, USA). The RNA integrity was checked by an Agilent 2100 Bioanalyser with RNA 6000 Nano Kits (Agilent, USA) with an RNA integrity number (RIN) more than 8. Small RNA libraries were prepared from 1 μg total RNA for each sample using the NEB Next, Multiplex Small RNA Library Prep Set kit for Illumina (NEB, USA) according to standard protocols. Briefly, the 3′ adapters and 5′ adapters were ligated to the 18–30 nt small RNAs selected by 15% PAGE gels from the total RNA. Several procedures were performed, including reverse transcription, and PCR amplification and purification, for constructing the small RNA libraries. Total RNA was purified to remove ribosomal RNA (rRNA) using the Ribo-zero rRNA Removal Kit (NEB, USA). The concentration and quality of the cDNA libraries was assessed by a Qubit 2.0 Fluorometer with the Qubit dsDNA HS Assay Kit (Life Technologies, USA) and an Agilent 2100 Bioanalyser with the High Sensitive DNA kit (Agilent, USA). Nine small RNA libraries and 9 long RNA libraries were generated for high-throughput sequencing using a HiSeq 4000 platform, yielding 50-bp single-end reads and 100-bp paired-end reads, respectively.

### Long RNA sequencing data analysis

FastQC (Version 0.11.5; http://www.bioinformatics.babraham.ac.uk/projects/fastqc/) and Trimmomatic (Version 0.36; default setting except MINLEN:50) programs were used in the paired-end mode for obtaining high-quality reads through trimming the low-quality reads (Q20) and contaminating 5′ and 3′ adapters reads [35]. The resulting high-quality reads were aligned using TopHat2 to the latest referenced Ensembl chicken genome (*Gallus gallus* 5.0) with paired-end mode and fr-firststrand options [36]. Then, the paired-end mapped reads were proceeded by the featureCounts (Version 1.5.1) program for counting the number of fragments per gene per library [37]. In this study, only known coding genes were selected for further analysis such as differentially expressed gene analysis and functional annotation.

### Small RNA sequencing data analysis

The raw data of miRNA sequencing was first proceeded by FastQC (Version 0.11.5; http://www.bioinformatics.babraham.ac.uk/projects/fastqc/), and FASTX-Toolkit (Version 0.0.13; http://hannonlab.cshl.edu/fastx_toolkit/index.html) programs were used with the single-end mode for obtaining high-quality reads through filtering low-quality reads (Q20), contaminating adapters and longer (> 23 nt) or shorter (< 18 nt) reads. Then, the high-quality reads were proceeded by three steps to identify miRNAs. Firstly, Blast software (version 2.2.31) was used to annotate the ncRNAs based on Rfam database (version 12.0) and removed rRNA, scRNA, snoRNA, snRNA, tRNA and other ncRNAs with E-value at 0.01. Then the output reads were aligned to the latest referenced Ensembl chicken genome (*Gallus gallus* 5.0) and available miRNA dataset (miRBase 21; http://www.mirbase.org/) by using BWA software (version 0.6) subsequently with one mismatch across the entire reads [38]. SAMtools (Version 1.4) was subsequently utilized to count the amounts of read numbers of all the miRNAs detected in each sample [39].

### Differentially expressed miRNAs and mRNAs

To identify differentially expressed miRNA and mRNA (DEGs), DESeq2 [40], which is based on the negative binomial distribution, was employed to normalize and evaluate the significant changes in the comparisons of F1 vs. N1, L2 vs. N1, and F1 vs. L2. DEGs of each comparison were selected with a setting of q value < 0.01 and fold change > 2 for further analysis.

### Validation of differentially expressed miRNAs and mRNAs by qPCR

To validate the results of high-throughput sequencing data, qPCR was performed to detect the expression patterns of altered miRNAs and mRNAs among each group. Among 33 altered miRNAs, there were 11 miRNAs overlapped between the comparisons of F1 vs. N1 and L2 vs. N1. About half of these overlapped miRNAs were randomly chosen for validation by qPCR. For the confirmation experiment of mRNA expression data, three genes were randomly chosen from overlapped mRNAs with differently expression patterns between the comparison of F1 vs. N1 and L2 vs. N1. Besides, another two reported candidate genes, PRLR and SPEF2, were also used for validation by qPCR. First, total RNA was used to generate the first strand cDNA library according to the stem-loop primer method for miRNA and the random primer method for mRNA using the PrimeScript™ RT reagent Kit (Takara, Japan). U6 and β-actin were used as reference genes for miRNA and mRNA testing, respectively. All the primers are listed in Additional file 1. The 20-μl PCR reaction solution was mixed according to the standard protocol for the SYBR Premix Ex Taq™ II kit (Takara, Japan), including 10 μl SYBR Premix Ex Taq™ II (2×), 0.8 μl forward primer, 0.8 μl reverse primer, 0.4 μl ROX Reference Dye II (50×), 2.0 μl cDNA and 6.0 μl H_2_O. The PCR reaction was performed on an ABI 7500 system (ABI, USA) as follows: 5 min at 95 °C for initial denaturation, then 35 cycles at 95 °C for 5 s, 57 °C for 30 s and 72 °C for 34 s, finally followed by the dissociation stage (95 °C for 15 s, 60 °C for 1 min and 95 °C for 15 s, then 60 °C for 15 s). The results were calculated and analysed by the 2^-ΔΔct^ method, in which ∆∆Ct = ∆Ct (target gene) – ∆Ct (reference gene) [41]. All data were obtained by repeating experiments three times. Student’s *t*-test was used to compare expression levels among different groups. The threshold for significance was set at *p* value < 0.05 and for high significance was set at p value < 0.01.

### miRNA target genes and bioinformatic analysis

MiRNA regulation mostly occurs through the interaction of miRNA and mRNA 3’UTR regions. We first downloaded all the chicken UTR sequences from BioMart (http://www.ensembl.org/biomart/martview/). Then, both RNAhybrid (https://bibiserv.cebitec.uni-bielefeld.de/rnahybrid/) and miRanda (http://www.microrna.org/microrna/home.do) software systems were used for predicting the targets of differentially expressed miRNAs [42, 43]. The seed sequences were of complete complementary base pairing without the G:U base pair, and the minimum free energy of the RNA secondary structure was less than − 20 kcal/mol. Only the overlapped targets identified by RNAhybrid and miRanda were selected for functional analysis.

In this study, thousands of altered genes and dozens of altered miRNAs were identified in the comparisons of F1 vs. N1 and L2 vs. N1. Comparing to N1 group with no feathering throw out, F1 and L2 indicates different feathering growth status with longer or shorter primary feathering. To understand the functional mechanism of these altered genes and miRNAs on feathering development, the differently expressed genes or putative target genes of differently expressed miRNAs generated from each paired comparison among F1, L2 and N1 groups were used for Gene Ontology and KEGG pathway enrichment by DAVID 6.7 (http://david.abcc.ncifcrf.gov/), respectively. KEGG results were filtered using *p* value < 0.05 based upon a Fisher Exact statistic methodology that previously described [44].

Since miRNA commonly acts as a negative regulator, up-regulated miRNA resulted in down-regulated target mRNA, and vice versa. Pearson’s correlation analysis was applied to identify the miRNA-mRNA pairs with inversed expression relationship from two independent data sets of miRNA and mRNA expression profiles in the F1, L2 and N1 groups.

## Additional file

Additional file 1: **Table S1.** Primers for qPCR. (DOCX 21 kb)

## Abbreviations

EF: Early-feathering
*ev*21: Avian endogenous virus 21
F1: Primary feathers are longer than primary-covert feathers up to 4.5 mm (mm)
L2: Primary feathers are shorter than primary-covert feathers
LF: Late-feathering
N1: Primary feathers do not extend out
PRLR: Prolactin receptor
qPCR: Quantitative real-time PCR
RPKM: Reads per kilobases per million reads
SPEF2: Sperm flagellar protein
TPM: Tag per million
