# Supplementary material for: Characterization of microRNA and mRNA expression profiles in skin tissue between early-feathering and late-feathering chickens
Source: BMC Genomics. 2018 May 25;19:399. doi: 10.1186/s12864-018-4773-z (PMC5970437; doi:10.1186/s12864-018-4773-z)
Supplement: Supplementary file 1 — Table S1. Primers for qPCR. (DOCX 21 kb) [file 12864_2018_4773_MOESM1_ESM.docx]

**Characterization of microRNA and mRNA expression profiles in skin tissue between early-feathering and** **late-feathering chickens**

Guijun Fang^1,2#^, Xinzheng Jia^1,2#^, Hua Li^1,3*^, Shuwen Tan^1,3^, Qinghua Nie^2^, Hui Yu^1,3^, Yin Yang^1^

^1^School of Life Science and Engineering, Foshan University, Foshan 528231, Guangdong, China

^2^College of Animal Science, South China Agricultural University, Guangzhou 510642, Guangdong, China

^3^Guangdong Tiannong Food Company Limited, Qingyuan 511827, Guangdong, China

^#^Equal contributors

^*^Correspondence author: okhuali@aliyun.com

**Table S1. Primers used in this study.**

|  | RT primer | |
| --- | --- | --- |
| miR-1552-5p | GTCGTATCCAGTGCAGGGTCCGAGGTATTCGCACTGGATACGACCACCCT | |
| miR-211 | GTCGTATCCAGTGCAGGGTCCGAGGTATTCGCACTGGATACGACAGGCAT | |
| gga-miR-365-2-5p | GTCGTATCCAGTGCAGGGTCCGAGGTATTCGCACTGGATACGACTCTGC | |
| miR-199b | GTCGTATCCAGTGCAGGGTCCGAGGTATTCGCACTGGATACGACACCAAA | |
| miR-6651-5p | GTCGTATCCAGTGCAGGGTCCGAGGTATTCGCACTGGATACGACCCCCTC | |
|  | **Forward Primer** | **Reverse Primer** |
| WNT3A | AGCAGAAGGGGTGAAGATTG | TGTCTAGCACAGGTCCGAAG |
| WNT8A | CTCAAAGTGGAGGAGAGGAG | CTATCTCCTGTGGCCTTTGT |
| PRLR | GCCCAGACTACAGAACATCA | GAGGATCCGAGCTGTTACTT |
| SPEF2 | ACACACCAGAACAGTGAAGC | AGGTCTGTAAAGGGCTGAAC |
| SOX17 | CAGCGAGTTCGAGCAGTA | GCGTAGCCGCAGTAGTAGAC |
| β-actin | GAGAAATTGTGCGTGACATCA | CCTGAACCTCTCATTGCCA |
|  | **Forward Primer** | **Reverse Primer** |
| miR-1552-5p | CTGCTTAGTGCGCGGTAAGCT | GTGCAGGGTCCGAGGT |
| miR-211 | GGCGTTCCCTTTGTCATCCT | GTGCAGGGTCCGAGGT |
| gga-miR-365-2-5p | GAGGGACTTTCAGGGGCAGC | GTGCAGGGTCCGAGGT |
| miR-199b | CGTGCACAGTAGTCTGCACA | GTGCAGGGTCCGAGGT |
| miR-6651-5p | CTGGAACCAGGTTGCCTAAG | GTGCAGGGTCCGAGGT |
| U6 | TAAGCCTGGACTGAGTAAGAGCG | CCATATTAGAAGCCCCTTTTTGT |
